# Use of online cultural content for mental health and well-being during COVID-19 restrictions: cross-sectional survey

**DOI:** 10.1192/bjb.2021.103

**Published:** 2022-10

**Authors:** Rebecca J. Syed Sheriff, Helen Adams, Evgenia Riga, Andrew K. Przybylski, Laura Bonsaver, Laura Bergin, Bessie O'Dell, Susan McCormack, Cathy Creswell, Andrea Cipriani, John R. Geddes

**Affiliations:** 1 University of Oxford, UK; 2 Oxford Health NHS Foundation Trust, UK; 3 University of Nottingham, UK

**Keywords:** Culture, museum, online, distress, mental health

## Abstract

**Aims and method:**

To gain a deeper understanding of the use of online culture and its potential benefits to mental health and well-being, sociodemographic characteristics and self-reported data on usage, perceived mental health benefits and health status were collected in an online cross-sectional survey during COVID-19 restrictions in the UK in June–July 2020.

**Results:**

In total, 1056 people completed the survey. A high proportion of participants reported finding online culture helpful for mental health; all but one of the benefits were associated with regular use and some with age. Reported benefits were wide-ranging and interconnected. Those aged under 25 years were less likely to be regular users of online culture or to have increased their use during lockdown.

**Clinical implications:**

There may be benefits in targeting cultural resources for mental health to vulnerable groups such as young adults.

Even before the COVID-19 pandemic, mental health was a major public health priority.^1^ Mental health problems have been exacerbated in the context of the COVID-19 pandemic^2,3^ and are set to remain a major public health concern, particularly among those most vulnerable, post-COVID-19.^4^

The first UK lockdown, initiated in March 2020, and subsequent restrictions forced the public indoors, leaving millions feeling isolated.^5^ Concurrently, the closure of cultural institutions such as museums, theatres and art galleries led to a rapid increase in both the production of, and engagement with, online culture. For example, in less than a month after it closed owing to the pandemic, the Metropolitan Museum of Art in New York reported a 4106% growth in streaming viewership, while the Ashmolean Museum in Oxford saw a huge increase in social media followers from March to July 2020, initiating the ‘beginning of a new era’ of virtual engagement for cultural institutions.^6^ With isolation being one of the biggest concerns during lockdown,^7^ many cultural institutions sought to find ways to bring people together to improve mental well-being. As Ernesto Ottone Ramírez, Assistant Director-General for Culture at UNESCO, said:

‘At a time when billions of people are physically separated from one another, culture has brought us together, keeping us connected and shortening the distance between us. It has provided comfort, inspiration and hope at a time of enormous anxiety and uncertainty.’^8^

Currently, there is no consensus on a single definition of well-being. There is general agreement that the concept includes the presence of positive emotions and moods (such as contentment and happiness), the absence of negative emotions (such as depression and anxiety), satisfaction with life, fulfilment and positive functioning, at a minimum.^9^ There are controversies in defining the term mental health, although one survey^10^ reported the preferred definition to be: ‘the capacity of each and all of us to feel, think, and act in ways that enhance our ability to enjoy life and deal with the challenges we face. It is a positive sense of emotional and spiritual well-being that respects the importance of culture, equity, social justice, interconnections and personal dignity’.^11^

The concept that culture and the arts are good for us is not new.^12^ Evidence for this view pervades the history of ideas dating back to Aristotle, who wrote that it is the natural instinct of all to ‘delight in works of imitation’.^12,13^ Kant described how the enjoyment of art can enhance personal well-being through pleasurable experience,^12^ and Schopenhauer describes art as one of the few means of protection from the anguish brought about by the unbearable nature of the human condition.^12,14^ Although there is a general academic view that culture has a positive effect on health and well-being,^15,16^ there have been long-standing debates regarding potentially adverse effects, including issues of inequitable provision and appreciation. This includes Bourdieu's theories of how cultural tastes originate in and perpetuate social stratification and thus may reinforce social hierarchies.^17^

However well-established these concepts of the positive effects of culture consumption are in the collective consciousness, there are significant research gaps regarding the effects of the arts and culture on health. Many studies have demonstrated correlations between cultural engagement and improved mental and physical health and longer life expectancy, but most are confounded by the sociodemographic determinants of health.^9^ More bluntly put, owing to inequitable access,^18,19^ those most likely to use culture and the arts are those at lower risk of poor health outcomes by virtue of characteristics such as income, education and ethnicity.^9^ Consequently, at the population level those least likely to be accessing these resources may be those most likely to benefit from them. It is also possible that the mechanisms by which those in particular sociodemographic groups have improved health outcomes relate to the richness of existence offered by such experiences.

Another flaw in much of the literature in this area has been such broad and non-specific definition of arts and culture as to obfuscate interpretation of the mental health effects.^9^ In the present paper, online cultural content refers to online resources from cultural institutions and includes museums, theatres, art galleries, libraries, archives and natural heritage organisations. In an attempt to avoid the pitfalls of casting too wide a net, this is a narrower definition than that adopted by others,^20^ which can also include reading, films and gaming.^20^

Much of the published work in the area of cultural engagement and mental health is in the form of community-based projects or pilot schemes for users of health services. Many of these involve social prescribing visits to cultural institutions^21^ in clinical populations.^22–25^ However, while the evidence regarding potential benefits of the arts on mental health in clinical populations is growing,^15,26–32^ a substantial body of evidence suggests that people with the greatest mental health need often have the poorest access to health services.^33^ Thus, concentrating research on clinical populations and social prescribing initiatives^34^ risks missing major opportunities to reduce health inequalities at the population level and may even compound such inequalities. In addition, it also represents a missed opportunity for prevention of mental illness and self-management of mental health.

## Aim and objectives

In light of the accelerated pace with which cultural institutions had expanded their online presence during the COVID-19 pandemic and building on the foundations of work to identify the health value of engaging with culture and the arts, we set out to describe who was using online cultural content, the perceived mental health benefits and the self-reported mental health of people using online culture during this period. To this end we conducted an online cross-sectional survey between 17 June and 22 July 2020. During that time the UK was not under full lockdown, but social distancing measures were in place and UK museums were closed.

Given the paucity of studies in this area, the aims of this study were primarily exploratory, to describe users of online culture, factors associated with being a regular user and increased engagement during the COVID-19 pandemic, self-reported benefits and the mental health status of this population.

## Method

### Ethical approval

The authors assert that all procedures contributing to this work comply with the ethical standards of the relevant national and institutional committees on human experimentation and with the Helsinki Declaration of 1975, as revised in 2008. All procedures involving human participants were approved by the Medical Sciences Interdivisional Research Ethics Committee of the University of Oxford (approval reference R70187/RE001-3).

### Procedure

We recruited 1056 participants aged 16 and over in the UK (or 18 and over, overseas) for the initial survey between 17 June and 22 July 2020. Participants were recruited through Facebook adverts, a press release, a pop-up advert that appeared on the Ashmolean Museum website, as well as the Ashmolean's public relations avenues (e.g. Twitter and a newsletter) and student unions. To enter the survey, participants followed a link to e-consent procedures (web traffic to the survey is shown in supplementary Table 1, available at https://doi.org/10.1192/bjb.2021.103). Of the 1056 participants, 500 chose to enter their email address and consented to be contacted again for further research. These people were emailed a link to e-consent to participate in a further free-text survey and 176 participants completed this survey between 10 and 30 July 2020.

### Measures

The initial survey included items on sociodemographics, usage and changes of use of online cultural content, health status and items on how online culture was perceived to affect on mental health and well-being. Sociodemographic items included age, gender, ethnicity, highest educational attainment, household income and the number of people living in the household. Questions relating to health status included COVID-19 isolation status, physical health problems and disability, as well as a measure of psychological distress.

A wide range of potential ways in which online cultural content could be helpful for mental health were offered as options, as well as an option with free-text boxes inviting participants to describe other ways online culture could be helpful for mental health. Items were also included comparing online with in-person experiences and whether benefits would be improved by including other people in the experience. The separate free-text survey was formulated to gain richer data on which and how cultural content could benefit mental health online or in person (if different from online).

Current self-reported psychological distress was measured in the initial survey using the Kessler Psychological Distress Scale (K10). The K10 strongly discriminates between community cases and non-cases of mental disorder identified by a structured clinical interview.^35^ The K10 comprises ten questions inquiring about the frequency of depressive and anxiety symptoms over the previous 4 weeks. Each item is rated on a 5-point Likert scale (1, none of the time; 5, all of the time) and scores are added to give a possible range of 10–50, with higher scores reflecting higher levels of psychological distress. The K10 is one of the most widely used mental health screening instruments^36^ and demonstrates good properties with regard to validity, reliability^36^ and sensitivity to change.^37^ A K10 score ≥20 was defined as clinically significant distress.^35,36^

### Statistical analysis

Data were imported into Stata v16.0 and descriptive statistics were used to describe the sociodemographic characteristics of the population. Percentages (and 95% confidence intervals) were calculated for population characteristics such as age, gender, income, education, ethnicity, location, employment status, household size, mental and physical health and isolation status, as well as online culture usage.

We used logistic regression techniques to analyse associations between sociodemographic and health variables and regular use of online culture (once a month or more) and increased engagement with online cultural content during COVID-19 restrictions. We described the percentage reporting specific mental health benefits of online culture. We used logistic regression techniques to examine the association of these with regular engagement and age group.

Logistic regression techniques were performed to analyse associations between clinically significant psychological distress, defined as scoring ≥20 on the K10,^36,38^ and sociodemographic and individual characteristics.

### Free-text analysis

Participants’ free-text responses for both surveys were pseudo-anonymised, with each response assigned a unique ID. The free-text responses were imported into NVivo 12 software for MacOS (QSR International, www.qsrinternational.com) to facilitate organisation and analysis of data. Two researchers (L.B. and B.O'D.) qualitatively analysed the free-text data by independently performing an inductive thematic analysis,^39^ allowing prevailing themes from the collected free text to be identified and subsequently analysed. All free-text responses were coded into themes, providing an initial framework for the qualitative data, with multiple allocations possible. This initial qualitative framework was then discussed collaboratively by the team to discern patterns in the data. The framework continued to be developed and refined to reflect the themes emerging from the free text, as well as the relationships between themes.

## Results

In total, 1056 participants completed the initial survey (between 17 June and 22 July 2020). High proportions of participants were over 44 years of age, female, had a university degree, reported being of White (British, Irish or Other) ethnic background and living with at least one other person. Of those who reported household income, the majority had a household income over £30 000 per year (Table 1).

**Table 1:** Population characteristics (*n* = 1056)

			95% CI	
	*n*	%	Lower	Upper
Age group, years				
16–24	77	7.4	5.9	9.1
25–34	73	7.0	5.6	8.7
35–44	88	8.4	6.9	10.3
45–54	152	14.6	12.5	16.8
55–64	294	28.2	25.5	31.0
65 and over	360	34.5	31.7	37.4
Gender				
Male	182	17.4	**15.2**	**19.9**
Female	855	81.9	79.4	84.1
Other	7	0.7	0.3	1.4
Prefer not to say	12	1.1		
Annual household income				
Less than £16 000	107	13.0	10.8	15.4
£16 000–£29 999	190	23.0	20.3	26.0
£30 000–£59 999	272	32.9	29.8	36.2
£60 000–£119 999	133	16.1	13.7	18.8
More than £120 K	124	15.0	12.7	17.6
Prefer not to say	230			
Highest educational attainment				
No qualification/vocational or school qualification	155	14.8	12.8	17.1
University degree	891	85.2	82.9	87.2
Prefer not to say	10			
Ethnicity				
Asian	18	1.7	1.1	2.7
Black	1	0.1	0.0	0.7
Chinese	7	0.7	0.3	1.4
White British, Irish, Other	961	91.0	89.1	92.6
Mixed-race White and Black	0	0.0		
Mixed-race Other	28	2.7	1.8	3.8
Middle Eastern	6	0.6	0.3	1.3
Other/prefer not to say	35	3.3	2.4	4.6
Location				
East Midlands	55	5.2	4.0	6.7
East of England	37	3.5	2.5	4.8
Greater London	88	8.3	6.8	10.2
North East England	22	2.1	1.4	3.1
Northern Ireland	4	0.4	0.1	1.0
North West England	50	4.7	3.6	6.2
Scotland	32	3.0	2.1	4.3
South East England	323	30.6	27.9	33.4
South West England	123	11.6	9.8	13.7
Wales	22	2.1	1.4	3.1
West Midlands	69	6.5	5.2	8.2
Yorkshire and the Humber	26	2.5	1.7	3.6
Outside of UK	205	19.4	17.1	21.9
Type of area				
City	392	37.3	34.5	40.3
Large town	173	16.5	14.3	18.8
Small town	222	21.1	18.8	23.7
Village	210	20.0	17.7	22.5
Rural (hamlet or isolated dwelling)	53	5.0	3.9	6.6
Missing	6			
Household				
Living alone	241	22.8	20.4	25.5
Living with one other	629	59.6	56.6	62.5
Living with more than one other	186	17.6	15.4	20.0
Previous COVID-19 infection				
No	933	88.4	86.4	90.2
Yes or suspected	122	11.6	9.8	13.6
Missing	1			
Isolation status				
Full isolation	76	7.3	5.8	9.0
Restricted	643	61.4	58.4	64.3
Leaving more often	329	31.4	28.6	34.3
Missing	8			
Employment status				
Full-time education	79	7.6	6.1	9.3
Employed	441	42.2	39.2	45.2
Unemployed/unwell/furlough/homemaker	128	12.2	10.4	14.4
Retired	398	38.0	35.1	41.0
Prefer not to say	10			
Physical health problem/disability				
No	834	80.3	77.7	82.6
Yes	205	19.7	17.4	22.3
Prefer not to say	17	1.6		
Mental health problems				
No	872	84.0	81.6	86.1
Yes	166	16.0	13.9	18.4
Prefer not to say	18			
Kessler Psychological Distress Scale score				
<20	583	56.0	53.0	59.0
20–24	217	20.8	18.5	23.4
25–29	123	11.8	10.0	13.9
≥30	118	11.3	9.5	13.4
Missing	15			
Change in use of online culture since initiation of COVID-19 restrictions				
Increased	753	71.3	68.5	74.0
Same	241	22.8	20.4	25.5
Less	62	5.9	4.6	7.5
Use of online culture				
Daily	247	23.4	20.9	26.0
Once a week or more	432	40.9	38.0	43.9
Once a month or more	176	16.7	14.5	19.0
Less than once a month	201	19.0	16.8	21.5
In-person museum use				
Visit in previous year	482	45.6	42.7	48.8

A high proportion of participants reported using online culture once a month or more (80.9%, 95% CI 78.5–83.2). This was associated with being older than 24, being female, having a household income of over £30 000 per year, having a university degree, being of White (British, Irish or Other) ethnicity and reporting an in-person museum visit in the previous year (Table 2).

**Table 2:** Associations between population characteristics and consumption of online cultural content^a^

Characteristic	Variable	OR of using online culture once a month or more	95% CI		OR of increased use of online culture in COVID-19 context	95% CI	
			Lower	Upper		Lower	Upper
Age group, years							
	16–24	1.00			1.00		
	25–34	**3.06**	**1.48**	**6.31**	1.59	0.82	3.06
	35–44	**3.85**	**1.88**	**7.87**	1.61	0.86	3.02
	45–54	**3.50**	**1.91**	**6.44**	**2.83**	**1.58**	**5.07**
	55–64	**4.05**	**2.35**	**6.99**	**2.66**	**1.58**	**4.47**
	65 and over	**4.03**	**2.38**	**6.85**	**2.35**	**1.42**	**3.89**
Gender	Female	**1.64**	**1.12**	**2.38**	**1.86**	**1.33**	**2.59**
Annual household income	More than £30 000	**1.56**	**1.13**	**2.17**	**1.52**	**1.14**	**2.02**
Highest educational attainment:	Undergraduate or postgraduate degree	**1.64**	**1.11**	**2.41**	0.99	0.68	1.43
Ethnicity	White British, Irish and other	**1.69**	**1.05**	**2.73**	**1.59**	**1.02**	**2.46**
Country	UK	1.08	0.73	1.61	0.91	0.65	1.27
Type of area							
	City	1.00					
	Large town	0.81	0.52	1.26	1.23	0.82	1.82
	Small town	0.95	0.63	1.45	1.21	0.84	1.74
	Village	1.07	0.69	1.66	1.23	0.85	1.78
	Rural (hamlet or isolated dwelling)	1.29	0.58	2.85	1.41	0.73	2.73
Household	Live alone	1.11	0.76	1.61	0.93	0.68	1.27
Previous COVID-19 infection	Yes or suspected	0.69	0.44	1.07	0.80	0.53	1.20
Isolation status							
	Full isolation	1.00			1.00		
	Restricted	1.07	0.57	2.01	0.93	0.54	1.62
	Leaving more often	0.60	0.32	1.15	0.63	0.36	1.11
Employment status							
	Full time education	1.00			1.00		
	Employed	1.66	0.95	2.89	1.46	0.89	2.41
	Unemployed/unwell/furlough/homemaker	1.35	0.70	2.61	**1.92**	**1.04**	**3.52**
	Retired	1.64	0.94	2.87	1.58	0.96	2.62
Physical disability	Yes	0.65	0.45	0.93	1.14	0.81	1.61
Mental health problems	Yes	0.75	0.50	1.12	0.80	0.56	1.14
K10 score	>19	0.78	0.58	1.07	1.09	0.83	1.42
In-person use	Visit in previous year	**1.76**	**1.28**	**2.43**	**1.57**	**1.19**	**2.06**

K10, Kessler Psychological Distress Scale.

a. Bold denotes significant difference identified based on odds ratio.

In addition, a high proportion of participants reported increasing their use of online culture since the initiation of COVID-19 restrictions (71.3%, 95% CI 68.5–74.0). Increased use was associated with being over 45, being female, having a household income of over £30 000 per year, being of White (British, Irish or other) ethnicity and being unemployed, unwell, on furlough or a homemaker and reporting an in-person visit in the previous year (Table 2).

A high proportion of participants reported that online cultural content was helpful for mental health and well-being. All bar one of the 15 most frequently reported mental health benefits were significantly associated with regular use of online culture (Table 3). In addition, there were significant differences in the benefits reported according to age, even when regular of use of online culture was controlled for. There were lower odds of people aged 25–34 reporting ‘feeling connected’ than other age groups (adjusted OR = 0.37, 95% CI 0.15–0.87) and lower odds of those in lower age groups reporting benefits due to adding structure to their day (aOR = 0.16, 95% CI 0.15–0.70). There were lower odds of reporting benefits due to increased personal productivity (aOR = 0.46, 95% CI 0.22–0.94) in those aged 55 and over compared with people aged 16–24.

**Table 3:** Associations between self-reported mental health benefits and regular use of online cultural content

Reported benefit	Responses, *n*	Respondents who reported the benefit			Association with regular use of online cultural content^a^		
		%	95% CI		OR	95% CI	
			Lower	Upper		Lower	Upper
Enjoyed a visual or aesthetic experience	996	94.58	92.98	95.83	13.0	7.1	23.8
Welcome distraction	954	92.03	90.13	93.59	5.0	3.0	8.1
Lifts mood	955	91.31	89.34	92.94	5.0	3.1	8.0
Engages the brain	922	88.72	86.51	90.61	5.6	3.6	8.6
Felt connected	909	82.07	79.43	84.43	4.8	3.2	7.0
Felt engaged	882	80.16	77.39	82.66	3.5	2.4	5.1
Personal productivity	863	72.54	69.46	75.42	3.0	2.1	4.3
Human endeavour	816	65.56	62.22	68.75	2.6	1.8	3.7
Reduce stress	843	65.48	62.2	68.62	2.4	1.6	3.4
Emotional outlet	735	52.38	48.76	55.98	1.6	1.1	2.3
Reduce loneliness	796	50.13	46.65	53.6	2.1	1.4	3.1
Add structure	797	48.93	45.47	52.41	2.6	1.7	3.8
Cope with uncertainty	760	34.21	30.91	37.67	1.5	1.0	2.3
Helped confidence	754	32.36	29.11	35.79	1.7	1.1	2.7
Insomnia	730	12.74	10.51	15.37	1.0	0.6	1.7

a. Regular use was defined as once a month or more.

Having a clinically significant level of distress (a K10 score of ≥20) was associated with being younger than 35, female, a household annual income below £30 000, belonging to a Black, Asian, Middle Eastern, mixed or other ethnic group, not having a university degree, being in full-time education, previous COVID-19 infection (diagnosed or suspected), self-isolating and having a mental or physical health problem/disability (Table 4). Reporting an in-person museum visit in the previous year had an inverse relationship with a clinically significant level of distress.

**Table 4:** Associations between population characteristics and a current clinically significant level of psychological distress (K10 > 19)^a^

Characteristic	Variable	K10 > 19		
		OR	95% CI	
			Lower	Upper
Age group, years				
	16–24	1.00		
	25–34	0.52	0.26	1.06
	35–44	**0.46**	**0.23**	**0.91**
	45–54	**0.32**	**0.17**	**0.60**
	55–64	**0.20**	**0.11**	**0.35**
	65 and over	**0.12**	**0.07**	**0.21**
Gender	Female	**2.07**	**1.46**	**2.93**
Annual household income	More than £30, 000	0.72	0.55	0.95
Highest educational attainment	University degree	**0.61**	**0.44**	**0.86**
Ethnicity	White, British, Irish and other	**0.60**	**0.40**	**0.92**
Country	UK	1.00	0.74	1.36
Type of area				
	City	1.00		
	Large Town	0.84	0.59	1.21
	Small Town	0.98	0.70	1.36
	Village	0.74	0.53	1.04
	Rural (hamlet or isolated dwelling)	0.59	0.32	1.08
Household				
	Living alone	1.00		
	Living with one other	1.20	0.88	1.62
	Living with more than one other	1.24	0.84	1.83
Previous COVID-19 infection	Yes or suspected	1.70	1.16	2.48
Isolation status				
	Full isolation	1.00		
	Restricted	**0.61**	**0.38**	**0.98**
	Leaving more often	**0.58**	**0.35**	**0.95**
Employment status				
	Full time education	1.00		
	Employed	**0.37**	**0.22**	**0.63**
	Unemployed/unwell/furlough/homemaker	**0.47**	**0.25**	**0.85**
	Retired	**0.15**	**0.09**	**0.26**
Physical health problem/disability	Yes	**1.83**	**1.34**	**2.49**
Mental health problem	Yes	**4.30**	**2.99**	**6.20**
In-person museum use	Visit in previous year	**0.66**	**0.52**	**0.84**

K10, Kessler Psychological Distress Scale.

a. Bold denotes significant difference identified based on odds ratio.

**Fig. 1:**
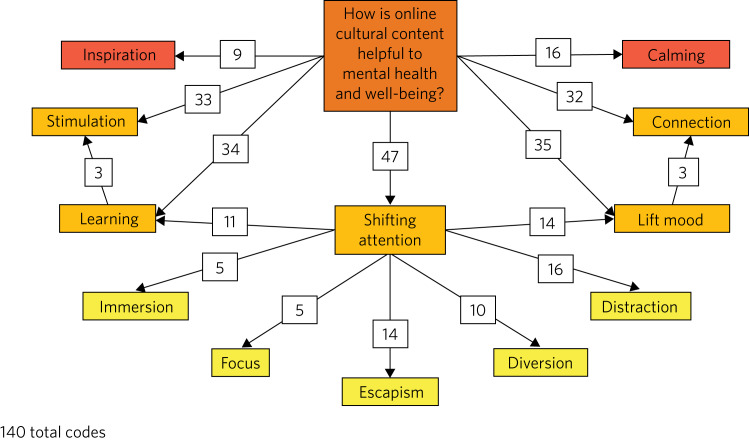
Main themes identified in responses to the survey question: ‘How is online cultural content helpful to mental health and well-being?’.

The free-text responses from the initial survey, along with the themes developing from the analysis of the free-text survey, were integrated with the quantitative data, providing the research team a more holistic understanding of the survey results. This process ensured reflexivity and enabled the research team to confirm the trustworthiness of the emerging themes. Figure 1 reflects the findings of the free-text survey, specifically regarding responses to the question ‘How is online cultural content helpful to mental health and well-being?’. Seven main themes emerged: inspiration, stimulation, learning, shifting attention, mood lifting, connection and calming. ‘Shifting attention’ was divided into the categories of immersion, focus, escapism, diversion and distraction, to provide a deeper analysis of the mental health effects gleaned from the shifting of participants’ attention. As Fig. 1 demonstrates, many of these categories are interrelated, a finding that reflects the multifaceted way in which the participants described that online cultural content affected mental health and well-being.

## Discussion

The self-reported mental health benefits of online cultural content in this study are broadly in keeping with a rich human history describing the mental health benefits of arts and culture.^12^ However, this survey suggests that the mental health benefits of online cultural content may currently be restricted to people within a narrow sociodemographic range. Almost all the self-reported mental health benefits are associated with regular use of online culture, and many groups at higher risk of mental health problems were least likely to have increased their use of online culture in the context of COVID-19. This suggests that inequitable engagement with cultural institutions is not being reduced by the transition to online forms of cultural engagement and that this transition may even worsen health disparities in the context of COVID-19. This is in urgent need of reversal and exploration of the potential synergies of online and in-person experiences as we emerge from COVID-19 restrictions.

The finding of overall increased use of online cultural content is broadly consistent with public perception and with another study that looked at online culture consumption more widely to include streaming music and movies as well as video games.^20^ The characteristics of people in our study reflect community cultural engagement in general, with high proportions being female, White, older^40^ and university educated. In addition, the overall profile broadly maps onto the usual onsite profile of museum users. Although this might indicate that cultural online resources are particularly helpful to this group, it seems likely that wider segments of the population might also derive benefit given equitable access to the content, as is the case with in-person engagement.^41^ This is particularly important given that those who are least likely to be engaging with cultural resources are those at higher risk of mental health problems during and in the aftermath of the COVID-19 pandemic, such as young people and ethnic minorities.^4^

We found that clinical levels of distress were associated with being young, female, lower income, lower educational attainment, previous COVID-19 diagnosis (confirmed or suspected), self-isolating, Black, Asian, Middle Eastern, mixed or other ethnic group, being in full-time education, and reporting a health problem. Although our population is not a representative population sample, these results echo epidemiological findings in the UK population more generally.^3,5^ In a lifestyle survey conducted in the UK in June 2020, rates of depression had almost doubled since before the pandemic, with high rates in young adults in particular.^5^ Although we used a different scale, which is designed also to detect anxiety, these associations are broadly similar.

A very high proportion of participants reported benefits to mental health from using online culture. The most commonly reported were ‘Enjoyed a visual experience or aesthetic experience’ and ‘Provided a welcome distraction’. The range and interconnection of these perceived benefits, and further elucidation of possible mechanisms such as learning and creativity, demonstrates the complexity and multifaceted nature of the relationship between mental health and cultural experiences which has been the subject of academic interest and debate throughout the history of ideas.^12^ Unlike in-person cultural experiences, the benefits of online experiences are not likely to be confounded by being in an outdoors environment or walking to, from and around the cultural venue, which introduces the confounder of gentle exercise.^9^ Further research is necessary to explore the longevity of the perceived changes, how they map onto the core components of common mental disorders and whether their benefits can be enhanced further, especially for those with the greatest mental health need.

Reported mental health benefits were more commonly reported by regular users of online culture, suggesting that online culture confers more mental health benefits in regular users. Our understanding of the potential synergies of different modes, as well as engagement with different types of cultural content, would benefit from further study and will be of particular significance as we emerge from COVID-19 restrictions.

Young people were not only less likely to be regular users of online culture, they were also less likely to have increased their use during the COVID-19 pandemic, despite having high rates of mental disorder in this context.^5^ This was also the case for people from Black, Asian, Middle Eastern, mixed or other ethnicities. Minority ethnic groups have been identified as being particularly vulnerable as we emerge from the COVID-19 pandemic^4^ and are amongst the most reluctant help-seekers.^42^

In addition, there was some evidence that the reported benefits vary with age. This suggests that young people may be missing out on the potential benefits of online culture. Further research is needed to elucidate whether this is due to preference or reduced awareness of these resources and their potential mental health benefits. In addition, in these younger age groups online access to such resources may improve accessibility especially for those from rural areas and disadvantaged groups. This suggests potential utility in strategies to enhance the mental health benefits according to age. This may have the added benefit of improved engagement with particularly underserved groups.

However, the view that online resources are universally accessible is erroneous. A range of demographic, educational, economic and skills-related factors merit attention when considering who might be able to benefit from the provision of online resources. National trends in internet use indicate that a number of determinants create a ‘digital divide’ which might limit who is able to access online cultural content.^43^ For example, those over the age of 50 are less likely to have regular access to the internet. Those with educational qualifications are more likely to use the internet: nearly 19 in 20 of those who have completed higher education are regularly online, whereas only 1 in 3 without qualifications have regular access to the internet. Household income is also important: nearly all UK households above the median (£30 000 per year) have access, compared with less than 2 in 3 households with lower income (£12 500). Finally, literacy skills remain a clear obstacle to regular internet use. More than 3 in 4 regular internet users are very confident in their reading skills, whereas less than 1 in 3 of those who do not use the internet say the same. The widespread availability of online cultural content should be kept in mind so that their study and development does not continue to reinforce existing social and economic inequalities.

### Strengths

This study took advantage of the world being in a state of flux, when the global COVID-19 pandemic meant that communities were driven indoors, which had an impact on levels of depression and anxiety^3,5^ at the population level. This cross-sectional survey was undertaken wholly during the time that UK cultural institutions had closed their doors. In such circumstances we were able to conduct a study of the use of online culture for mental health and well-being in unprecedented times. However, as a consequence we are lacking previous similar studies with which to compare our findings.

Of the 1056 participants, almost half gave their details for follow-up research, indicating considerable engagement with the subject. A high proportion of participants reported mental health benefits from use of online cultural content. We believe that this provides initial evidence that these resources may be a credible, valued and effective approach to improving mental health and that further research is warranted both to optimise and to reliably estimate potential benefits.

### Limitations

This survey used a convenience sampling strategy. This was essential in planning the study because of the time-limited nature of the initial restrictions. However, there are also clear disadvantages of this sampling strategy due to the intrinsic problem of the non-representativeness introduced by volunteer bias. In addition, as this survey was cross-sectional, the direction of these associations is unclear. It may be the case that using cultural content improves mental health, or alternatively, that people who enjoy cultural content both use it more regularly and recognise more benefits than others.

## Conclusions

These findings indicate that during a global pandemic that has had a catastrophic impact on mental health at the population level^3,5^ the move to online provision may have compounded pre-existing inequalities in access to cultural resources. Some of those with the greatest mental health needs as we emerge from the pandemic^4^ may be least likely to be benefitting from the potential mental health benefits of online culture.

Targeting cultural content to optimise mental health benefits for vulnerable groups such as young adults and ethnic minorities are a priority as we emerge from COVID-19 restrictions. In particular, it is important to identify barriers to engagement and develop interventions to increase engagement and optimise mental health benefits. These activities would benefit from taking place outside of the remit of social prescribing initiatives, as these vulnerable groups are often the least likely to seek help from healthcare services for their mental health.^44–46^ Access to online culture and potential synergies between online and in-person experiences provides a potential opportunities for optimisation. This study provides some initial indication that the development of cultural content for mental health would benefit from targeting by age group, enhancing the value of online cultural content as a potentially effective, acceptable and accessible non-clinical intervention for common mental health problems. Programmatic research is now required to target, develop and evaluate these resources further.

## Data Availability

Data is available from the authors upon reasonable request.

## About the authors

**Rebecca J. Syed Sheriff**, Senior Clinical Research Fellow, Department of Psychiatry, University of Oxford; Oxford Health NHS Foundation Trust, Warneford Hospital, Oxford; and Institute of Mental Health, University of Nottingham, UK. **Helen Adams,** Digital Engagement Lead, Gardens, Libraries and Museums, University of Oxford, UK. **Evgenia Riga**, Research Assistant, Department of Psychiatry, University of Oxford, UK. **Andrew K. Przybylski**, Director of Research, Oxford Internet Institute, University of Oxford, UK. **Laura Bonsaver**, Project Officer, Ashmolean Museum, University of Oxford, UK. **Laura Bergin**, DPhil student, School of Anthropology and Museum Ethnography, University of Oxford, UK. **Bessie O'Dell**, DPhil student, Department of Psychiatry, University of Oxford, UK. **Susan McCormack**, Director of Public Engagement, Ashmolean Museum, University of Oxford, UK. **Cathy Creswell**, Professor of Developmental Clinical Psychology, Department of Psychiatry, University of Oxford, UK. **Andrea Cipriani**, Professor of Psychiatry, Department of Psychiatry, University of Oxford, UK. **John R. Geddes**, Professor of Psychiatry, Department of Psychiatry, University of Oxford, UK.

## Supplementary material

For supplementary material accompanying this paper visit http://doi.org/10.1192/bjb.2021.103. click here to view supplementary material
